# Associations of leisure-time, occupational, and commuting physical activity with risk of depressive symptoms among Japanese workers: a cohort study

**DOI:** 10.1186/s12966-015-0283-4

**Published:** 2015-09-18

**Authors:** Keisuke Kuwahara, Toru Honda, Tohru Nakagawa, Shuichiro Yamamoto, Shamima Akter, Takeshi Hayashi, Tetsuya Mizoue

**Affiliations:** Department of Epidemiology and Prevention, Center for Clinical Sciences, National Center for Global Health and Medicine, 1-21-1 Toyama, Shinjuku-ku, Tokyo, 162-8655 Japan; Teikyo University Graduate School of Public Health, 2-11-1 Kaga, Itabashi-ku, Tokyo, 173-8605 Japan; Hitachi Health Care Center, Hitachi, Ltd., 4-3-16 Ohse-cho, Hitachi, Ibaraki 317-0076 Japan

**Keywords:** Cohort studies, Prevention, Depression, Dose of exercise

## Abstract

**Background:**

Leisure-time physical activity is associated with a lower risk of depression. However, the precise shape of the dose–response relationship remains elusive, and evidence is scarce regarding other domains of activity. We prospectively investigated associations of physical activity during leisure, work, and commuting with risk of depressive symptoms in Japanese workers.

**Methods:**

We conducted a cohort study of 29 082 Japanese workers aged 20–64 years without psychiatric disease (including depressive symptoms) at baseline with a maximum 5-year follow-up. Physical activity was self-reported. Depressive symptoms were assessed by 13 self-report questions on subjective symptoms. Hazard ratios (HRs) and 95 % confidence intervals (CIs) for incidence of depressive symptoms were calculated using Cox regression analysis.

**Results:**

During a mean follow-up of 4.7 years, 6177 developed depressive symptoms. Leisure exercise showed a U-shaped association with risk of depressive symptoms adjusting for potential confounders. Additional adjustment for baseline depression scores attenuated the association, but it remained statistically significant (P for trend = 0.037). Compared with individuals who engaged in sedentary work, the HR (95 % CI) was 0.86 (0.81, 0.92) for individuals who stand or walk during work and 0.90 (0.82, 0.99) for those who are fairly active at work. However, the association disappeared after adjusting for baseline depression scores. Walking to and from work was not associated with depressive symptoms.

**Conclusions:**

The findings suggest that leisure-time exercise has a U-shaped relation with depressive symptoms in Japanese workers. Health-enhancing physical activity intervention may be needed for individuals who engage in sedentary work.

## Background

Depression is a common mental disorder estimated to affect 350 million people worldwide [1]. It is associated with poor health outcomes including stroke [2], cardiovascular disease [3], type 2 diabetes [4], cancer mortality [5], and disability-adjusted life years [6], and thus, prevention of depression is a major global health issue. Physical activity is a lifestyle factor that might aid in prevention. A meta-analysis of cohort studies [7] showed that leisure-time physical activity is associated with a lower risk of developing depression. In addition, accumulating evidence [8] suggests that the currently recommended dose (150 min per week of moderate-intensity physical activity, 75 min per week of vigorous-intensity physical activity, or a combination of the two intensities) by the US government [9] and the World Health Organization (WHO) [10] can decrease risk of depression.

Several important issues need to be resolved. First, cohort studies on physical activity and depression have mainly been conducted in Western countries [7], and data are scarce in Asia [11]. Specifically, no prospective data on this issue are available in Japan. Given that Asians, including Japanese individuals, have a high suicide mortality rate [12], it is urgently necessary to elucidate protective factor against depression, which presents a high risk of suicide [13]. Second, the precise shape of dose-relationship between leisure-time physical activity and depressive symptoms remains elusive [14, 15]. Additional evidence on the dose–response relationship would help detect target individuals for physical activity interventions. Third, previous cohort studies on this issue focused on leisure-time physical activity alone or total physical activity (i.e., combination of leisure-time and occupational physical activity) [7]. Although few cross-sectional studies have investigated the association between other domains of physical activity, such as occupational [16–18] or commuting activity [16, 17], and depressive symptoms, no prospective studies have been conducted. To address these issues, we prospectively investigated the associations of leisure-time, occupational, and commuting physical activity with risk of depressive symptoms among Japanese workers using annual health checkup data.

## Methods

### Setting

The present study was performed on a subcohort of the Japan Epidemiology Collaboration on Occupational Health (J-ECOH) Study [19, 20], an ongoing, large-scale study among Japanese workers that includes more than 10 companies. The J-ECOH Study was announced in each company using posters. In Japan, workers are obliged to undergo health examination at least once a year under the Industrial Safety and Health Act; nearly all workers attend their health examination in each year. Participants did not provide their verbal or written informed consent to take part in the study but were given an opportunity to refuse the use of their data for research, according to the Japanese Ethical Guidelines for Epidemiological Research. The details of the J-ECOH Study have been described elsewhere [19, 20]. The study protocol was approved by the Ethics Committee of the National Center for Global Health and Medicine, Japan.

Of the participating companies in the J-ECOH Study, the present analysis included data from one company (electrical machinery and apparatus manufacturing) where detailed information on physical activity has been collected as a part of periodic health check-ups since 2006. Participants were 50 246 workers (41 039 men and 9207 women) aged 20–64 years in that company who underwent their health check-ups between April 2006 and March 2007 (baseline period).

### Participants

Of the eligible 50 246 workers, 16 412 were excluded due to missing data on depressive symptoms or history of psychiatric disease (*n* = 5654), having depressive symptoms defined as a depression score of 26 or higher (*n* = 10 152), or having history of psychiatric disease (*n* = 717), cardiovascular disease (*n* = 309), stroke (*n* = 99), or cancer (*n* = 376) at baseline. Of the remaining participants (*n* = 33 834), we excluded 1485 without data on physical activity (*n* = 790) or those who engaged in unspecified activity only during leisure (*n* = 703). Some of the participants met two or more exclusion criteria. Furthermore, we excluded individuals without data on covariates (*n* = 1810). Lastly, we excluded 1457 workers who did not attend any subsequent health check-ups or who had no measurement of depressive symptoms in a subsequent health check-up, leaving 29 082 workers (24 676 men and 4406 women), aged 20–64 years (mean: 42.7 years) for analysis.

### Assessment of physical activity

Participants were asked whether they regularly engaged in any physical activity during leisure. If yes, they were further asked to select up to three activities among a list of 20, together with the frequency (times per month) and duration of time per occasion (minutes) for each activity. If participants engaged in activities that were not listed in the questionnaire, they were instructed to choose an activity of similar intensity from the list.

Of the 20 regular exercise or sports activities, one activity named “other” was not used for further analysis. The value of metabolic equivalents (METs) for each activity was assigned according to a compendium of physical activities [21]. If the MET value of an activity was not listed in the compendium, we assigned the MET value of a similar activity. Of 19 activities, 13 (walking not for work or commuting, walking fast not for work or commuting, swimming, golf practice, golf, baseball, softball, cycling, table tennis, pang pong, badminton, muscle strength training, and radio gymnastics) were classified as moderate activity (3–6 METs). Six (light jogging [about 6 min/km], jogging, soccer, tennis, aerobics, and rope jump) were classified as vigorous activity (>6 METs). These were used to calculate the total MET hours of leisure-time exercise per week by multiplying the METs, duration, and frequency of the activity. These activities covered most activities that are common in Japan [22]. Participants were categorized into six groups according to the dose of leisure-time exercise per week: no exercise (0 MET hours), very low (0.1 to < 3.75 MET hours), low (3.75 to < 7.5 MET hours), medium (7.5 to < 16.5 MET hours), high (16.5 to < 25.5 MET hours), and very high (≥25.5 MET hours), in accordance with current physical activity guidelines [9, 10] and previous studies [23, 24].

Occupational physical activity was assessed by a single question with four response options (mostly sedentary, mostly standing, walking often, or fairly active). We combined the two categories of mostly standing and walking often (standing or walking at work) to increase statistical power [25]. Duration of walking to and from work (in minutes) was self-reported and categorized as < 20, 20 to < 40, and ≥ 40 min for the analysis. These cutoffs were determined based on previous Japanese studies [26, 27].

### Assessment of depressive symptoms

In the participating company, 40 items measuring subjective symptoms including 13 typical depressive symptoms were assessed by a self-reported questionnaire [28] at an annual health check-up. The depressive symptoms included in that questionnaire were similar to those asked in widely used questionnaires, including the Center for Epidemiologic Studies Depression Scale (CES-D) [29] and the Self-Rating Depression Scale (SDS) [30]. Respondents were asked to choose among four response options (“1 no,” “2 sometimes,” “3 often,” and “4 always”) for questions 1 to 12 and among three options (“1 no,” “2 yes,” and “3 severe”) for question 13 [28]. In order to assign an equal weight to all questions, scores of 2 and 3 for question 13 were adjusted to 2.5 and 4, respectively. The total depression score (ranging from 13 to 52 points) was then calculated as the sum of the scores across the questions if participant completed all 13 questions. This score is highly correlated with that of the SDS (Correlation coefficient = 0.752) among Japanese workers [28]. In addition, internal consistency of the depression score was high; Cronbach’s alpha was 0.887.

Recent studies of Japanese workers have shown that the prevalence of depressive symptoms is about 20–30 % [31, 32]. Therefore, we defined depressive symptoms as scores in the fourth quartile of depression scores among the total study population at baseline. According to the definition, individuals with a depression score of 26 or more are considered as having depressive symptoms.

### Assessment of other variables

Body height was measured to the nearest 0.1 cm and body weight to the nearest 0.1 kg. Body mass index (BMI) was calculated as weight in kg divided by squared height in m. History of disease, subjective symptoms, work-related factors, marital status, health-related lifestyle including physical activity and alcohol were ascertained using a standard questionnaire. Job position was categorized as high (department chief, department director, or higher position) or low (others). Smoking status (never, past, or current) and, for current smokers, the number of cigarettes smoked per day were asked. Total amount of alcohol consumption was calculated using data on frequency (number of days per week) and amount of alcohol consumption of common beverages (Japanese sake; beer; whiskey; *shochu*, which is a Japanese distilled beverage; *chuhai*, which is a sweetened beverage mixed with *shochu*; and wine) per day, as indicated by an equivalent amount of one unit (*go*) of Japanese sake. One *go* of Japanese sake contains approximately 23 g of ethanol.

### Statistical analysis

Descriptive results of study population are expressed as means for continuous variables and percentages for categorical variables. Associations between leisure-time exercise dose and covariates were examined using regression analysis by assigning the median value for each category of leisure-time exercise dose to each exercise category and treating this variable as continuous. Person-time was calculated from the date of the baseline examination to the date of diagnosis of depressive symptoms at a subsequent examination or to the date of the last examination, whichever came first. Hazard ratios (HRs) and their 95 % confidence intervals (CIs) for the incidence of depressive symptoms associated with leisure-time, occupational, and commuting was estimated using Cox proportional hazards models. First, we adjusted for age (years, continuous), sex, BMI (<18.5, 18.5 to < 23, 23 to < 25, 25 to < 30, or ≥ 30 kg/m^2^), shift work (yes or no), job position (high or low), smoking status (non-smoker, current smoker consuming 1 to 10, 11–20, or ≥ 21 cigarettes per day), alcohol consumption (non-drinker, drinker consuming < 1, 1 to < 2, ≥ 2 *go* of Japanese sake equivalent per day), sleep duration (<5, 5 to < 6, 6 to < 7, or ≥ 7 h per day), marital status (unmarried, married, or divorced or bereaved) (model 1). In model 2, other types of physical activity were mutually adjusted. That is, occupational and commuting physical activity were adjusted for leisure-time exercise, leisure-time exercise and commuting physical activity were adjusted for occupational physical activity, and leisure-time exercise and occupational physical activity were adjusted for commuting physical activity. In model 3, we additionally adjusted for baseline depression score (continuous). Variance inflation factor, an indicator of multi-collinearity, was low (<1.4) for all the variables in model 3.

The trend association between leisure-time exercise and risk of depressive symptoms was assessed by assigning the median value in each category of leisure-time exercise with no leisure-time exercise as the reference. For the trend association in relation to occupational physical activity, we assigned ordinal numbers (1 to 3) to sedentary work, stand or walk during work, and fairly active work, respectively. For walking to and from work, we assigned the median value of each category of walking to and from work. We assessed the shape of the relationship between leisure-time exercise and incidence of depressive symptoms using a cubic spline regression with three knots based on the current physical activity guidelines.[9, 10] The reference value for estimating the HRs (95 % CIs) was chosen as 0 MET hours per week of leisure-time exercise. As a sensitivity analysis, we repeated the main analysis for leisure-time exercise, occupational physical activity, and commuting physical activity after exclusion of participants with short follow-up term (<2 years). We tested the proportional-hazards assumption with the Schoenfeld residuals. We found no significant deviations for all covariates except for marital status. Two-sided P-values < 0.05 were considered as statistically significant. All analyses were performed with Stata version 13.1 (Stata Corp, College Station, Texas).

## Results

At baseline, participants in the main analysis (*n* = 29 082) had lower baseline depression score (18.5 *vs.* 26.1) and were likely to engage in regular exercise during leisure (37.1 % *vs.* 33.4 %) and walk to and from work compared with excluded participants (*n* = 21 164). They were older (42.7 years *vs.* 39.9 years) and less likely to be female (15.2 % *vs.*22.7 %) and unmarried (28.8 % *vs.* 40.0 %) than those who were excluded. Those included were in a higher position and worked shorter hours. Other variables including BMI, smoking, shift work, occupational physical activity, and alcohol use were not materially different between the two groups.

The characteristics of participants according to the dose of leisure-time exercise are shown in Table 1. Participants who engaged in more exercise during leisure tended to be male and young, engage in sedentary work, have a higher job position, and were less likely to smoke and drink alcohol, get married, or engage in shift work and walking for commuting to and from work. BMI did not differ by the dose of leisure-time exercise.

**Table 1:** Baseline characteristics of participants according to the dose of leisure-time exercise

	Dose of leisure-time exercise						
	No exercise	Very low	Low	Medium	High	Very high	P for trend^a^
No. of participants	17 704	2753	2627	3422	1284	1292	
Male sex	14 608 (82.5)	2355 (85.5)	2321 (88.4)	3049 (89.0)	1168 (91.0)	1175 (90.9)	<0.001
Age, year	43.1 ± 10.2	40.7 ± 10.6	42.1 ± 10.9	42.7 ± 11.1	43.5 ± 11.0	42.4 ± 12.2	0.28
BMI, kg/m^2^	23.3 ± 3.4	23.3 ± 3.4	23.5 ± 3.3	23.6 ± 3.1	23.5 ± 3.1	23.3 ± 3.0	0.002
Baseline depression score	18.9 ± 3.7	18.4 ± 3.7	18.2 ± 3.7	18.1 ± 3.7	17.9 ± 3.6	17.6 ± 3.8	<0.001
Smoking	7666 (43.3)	1170 (42.5)	1040 (39.6)	1345 (39.3)	469 (36.5)	483 (37.4)	<0.001
Heavy alcohol drinking^b^	1517 (8.6)	188 (6.8)	191 (7.3)	271 (7.9)	120 (9.4)	106 (8.2)	0.91
Married	12 728 (71.9)	1882 (68.4)	1843 (70.2)	2429 (70.1)	941 (73.3)	871 (67.4)	0.02
High job position	2970 (16.8)	397 (14.4)	469 (17.9)	727 (21.2)	307 (23.9)	181 (14.0)	0.005
Shift work	3394 (19.2)	612 (22.2)	439 (16.7)	567 (16.6)	189 (14.7)	232 (18.0)	<0.001
Long overtime work (≥45 h/month)	5736 (32.4)	843 (30.6)	827 (31.5)	1094 (32.0)	365 (28.4)	337 (26.1)	<0.001
Sedentary work	10 284 (58.1)	1554 (56.5)	1599 (60.9)	2174 (63.5)	815 (63.5)	699 (54.1)	0.20
<20 min of walking to and from work	9487 (53.6)	1613 (58.6)	1400 (53.3)	1876 (54.8)	733 (57.1)	730 (56.5)	0.011
Dose of exercise, MET hours per week	0	>0 to < 3.25	3.25 to < 7.5	7.5 to < 16.5	16.5 to < 25.5	≥25.5	

Data are shown as mean ± standard deviation for continuous variables and number (percentages) for categorical variables

*BMI* body mass index, *MET* metabolic equivalent

^a^P for trend was calculated using linear regression analysis for continuous variables and logistic regression analysis or categorical variables

^b^ ≥ 2 go of Japanese sake equivalent, 1 go of Japanese sake contains approximately 23 g of ethanol

During a mean follow-up of 4.7 years with 135 747 person-years, 6177 developed depressive symptoms. Table 2 shows the association of leisure-time, occupational, and commuting physical activity with risk of developing depressive symptoms. Exercise during leisure showed an inverse association with risk of depressive symptoms. The multivariable-adjusted HRs (95 % CIs) were 1 (reference), 0.90 (0.82, 0.98), 0.82 (0.76, 0.89), 0.72 (0.63, 0.83), and 0.80 (0.70, 0.91) for 0, > 0 to < 3.75, 3.75 to < 7.5, 7.5 to 16.5, 16.5 to 25.5, and ≥ 25.5 MET-hour per week of leisure-time exercise, respectively (P for trend < 0.001), adjusting for potential confounders, including occupational and commuting physical activity (model 2). Additional adjustment for baseline depression score slightly attenuated the association, but it remained statistically significant (P for trend = 0.031). After exclusion of participants with <2 years of follow-up term (4,966 participants including 3,069 incident cases of depressive symptoms), the hazard ratios were not materially changed, although the association became non-significant (P for trend = 0.078).

**Table 2:** Risk of developing depressive symptoms according to domains of physical activity

	Unadjusted	P for trend	Model 1^a^	P for trend	Model 2^b^	P for trend	Model 3^c^	P for trend
Leisure-time exercise								
MET hours per week								
0 (*n* = 17 704)	1		1		1		1	
>0 to < 3.75 (*n* = 2753)	0.98 (0.90, 1.06)		0.89 (0.82, 0.97)		0.90 (0.82, 0.98)		0.97 (0.89, 1.05)	
3.75 to < 7.5 (*n* = 2627)	0.88 (0.81, 0.97)		0.84 (0.77, 0.92)		0.84 (0.77, 0.92)		0.90 (0.82, 0.98)	
7.5 to 16.5 (*n* = 3422)	0.85 (0.78, 0.92)		0.82 (0.76, 0.90)		0.82 (0.76, 0.89)		0.92 (0.84, 0.99)	
16.5 to < 25.5 (*n* = 1284)	0.73 (0.64, 0.84)		0.72 (0.63, 0.83)		0.72 (0.63, 0.83)		0.85 (0.74, 0.98)	
≥25.5 (*n* = 1292)	0.85 (0.74, 0.96)	<0.001	0.79 (0.69, 0.90)	<0.001	0.80 (0.70, 0.91)	<0.001	0.95 (0.83, 1.08)	0.031
Occupational physical activity								
Sedentary (*n* = 17 125)	1		1		1		1	
Standing or walking (*n* = 9579)	0.88 (0.84, 0.93)		0.86 (0.81, 0.92)		0.86 (0.81, 0.92)		0.98 (0.92, 1.04)	
Fairly active (*n* = 2378)	0.96 (0.88, 1.05)	0.002	0.90 (0.82, 0.99)	<0.001	0.91 (0.82, 1.00)	<0.001	1.01 (0.91, 1.12)	0.81
Walking to and from work, min								
0 to < 20 (*n* = 15 839)	1		1		1		1	
20 to < 40 (*n* = 9180)	0.98 (0.93, 1.04)		1.02 (0.96, 1.08)		1.01 (0.95, 1.07)		1.01 (0.95, 1.07)	
≥40 (*n* = 4063)	0.92 (0.86, 0.99)	0.053	0.97 (0.90, 1.05)	0.74	0.95 (0.88, 1.03)	0.38	1.03 (0.95, 1.11)	0.54

Data are shown as hazard ratios (95 % confidence intervals)

^a^Adjusted for age (year, continuous), sex, body mass index (<18.5 kg/m^2^, 18.5 to < 23 kg/m^2^, 23.0 to < 25.0 kg/m^2^, 25.0 to < 30.0 kg/m^2^, or ≥ 30.0 kg/m^2^), smoking (non-smoker, smoker consuming 1 to < 10, 10–20, or ≥ 20 cigarettes per day), alcohol consumption (non-drinker, drinker consuming < 1, 1 to < 2, or ≥ 2 *go* of Japanese sake equivalent per day, where 1 *go* of Japanese sake contains approximately 23 g of ethanol), shift work (yes or no), overtime work (<45 h per month, 45 to < 60 h per month, 60 to < 80 h per month, 80 to < 100 h per month, or ≥ 100 h per month), job position (high or low), marital status (unmarried, married, or divorced or bereaved)

^b^Additionally adjusted for mutual relations (e.g., adjustment for occupational and commuting activity for leisure-time exercise)

^c^Further adjusted for baseline depression scores (continuous)

**Fig. 1:**
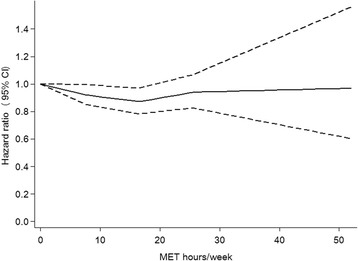
Restricted cubic spline regression for the association between leisure-time exercise and risk of depressive symptoms. Footnote: Knots were placed in accordance with the current physical activity guidelines by the World Health Organization. The reference value is 0 MET hours of leisure-time exercise per week. The continuous line presents hazard ratios and the dashed line presents 95 % confidence intervals. The model was adjusted for age (year, continuous), sex, body mass index (<18.5 kg/m^2^, 18.5 to < 23 kg/m^2^, 23 to < 25 kg/m^2^, 25 to < 30 kg/m^2^, or ≥ 30 kg/m^2^), smoking (non-smoker, smoker consuming 1 to < 10, 10–20, or ≥ 20 cigarettes per day), alcohol consumption (non-drinker, drinker consuming < 1, 1 to < 2, or drinker consuming ≥ 2 *go* of Japanese sake equivalent per day, where 1 *go* of Japanese sake contains approximately 23 g of ethanol), shift work (yes or no), overtime work (<45 h per month, 45 to < 60 h per month, 60 to < 80 h per month, 80 to < 100 h per month, or ≥ 100 h per month), job position (high or low), marital status (unmarried, married, or divorced or bereaved), occupational physical activity (sedentary, standing or walking, or fairly active), commuting physical activity (<20 min, 20 min to < 40 min, or ≥ 40 min of walking to and from work), and baseline depression score (continuous)

Figure 1 shows a spline regression model comparing the dose of leisure-time exercise and depressive symptoms. The relationship between the leisure-time exercise and risk of depressive symptoms differed around 16.5 MET hours per week of leisure-time exercise. The risk was gradually decreased up to 16.5 MET hours per week of leisure-time exercise, while the risk turned to increase at 16.5 MET hours per week of leisure-time exercise.

As shown in Table 2, compared with individuals who engaged in sedentary work, the multivariable-adjusted HRs (95 % CIs) as 0.86 (0.81, 0.92) among individuals who stand or walk during work and 0.91 (0.82, 1.00) among workers who are fairly active during work in model 2 (P for trend < 0.001). The association disappeared after further adjustment for baseline depression scores (P for trend = 0.81). Walking to and from work was not associated with risk of depressive symptoms in any models (all Ps > 0.05). A sensitivity analysis excluding participants with <2 years of follow-up term showed that the results were not materially changed for occupational and commuting physical activity (data not shown).

## Discussion

In the present study of a large-scale Japanese working population, there was a U-shaped association between leisure-time exercise and risk of depressive symptoms, with the lowest risk reduction around 16.5 MET hours per week of leisure-time exercise. Sedentary work was associated with a higher risk of depressive symptoms; however, this association was diminished after adjusting for baseline depression scores. This is one of few Asian studies examining the relationship between physical activity and depressive symptoms and the first prospective study on the association between different domains of physical activity and depressive symptoms.

Quantifying the extent of risk reduction in depressive symptoms with reference to a well-known physical activity recommendation by the WHO [10] and the US government [9] is an important task. The present analysis showed that risk of depressive symptoms gradually decreased from no exercise to a high dose of leisure-time exercise (16.5 to < 25.5 MET hours per week, which approximately corresponds to double the dose of the currently recommended level). No additional reduction was observed at a very high exercise dose (above 25.5 MET hours per week). The shape of this association was supported by the cubic spline regression analysis, although caution is needed in the interpretation of results for very high dose of exercise, in which only 4.4 % workers engaged. In line with the present observations, a US cohort study [15] reported a U-shaped association between vigorous-intensity exercise and depressive symptoms, showing the greatest risk reduction (18 %) associated with 3–4 h per week of vigorous-intensity exercise. In an Australian study [14], for none to low physical activity, the risk of depressive symptoms decreased linearly, while the risk plateaued for moderate to high physical activity. Admitting limited data on the shape of association, the present results together with previous findings suggest that enhancing leisure-time physical activity can significantly contribute to the prevention of depressive symptoms given a high prevalence of physical inactivity in adults [33].

We found that sedentary work was associated with a significantly higher risk of developing depressive symptoms, possibly due to the higher depressive scores associated with sedentary work at baseline, as the association disappeared after adjusting for baseline depression scores. The present results suggest that sedentary work may not increase depression risk independently of baseline depressive state. The existing literature on this issue is limited, as there are no prospective studies and the few cross-sectional studies have provided inconsistent results [16–18]. An Australian study of women [18] showed that the prevalence of depressive symptoms was not increased among individuals who engaged in a higher dose of occupational physical activity. Another Australian study [16] in men but not women also reported no increased odds of depression associated with high levels of occupational physical activity. In contrast, an Estonian study of women [17] reported increased odds of having depressive symptoms for high occupational physical activity. Due to differences in measurement of occupational physical activity and depressive symptoms as well as characteristics of study participants, we cannot draw any conclusions from the current evidence. Given the paucity of prospective data on this issue, additional efforts are needed to determine the effect of occupational physical activity on depressive symptoms.

We failed to show an inverse association between walking to and from work and risk of depressive symptoms. This null finding is consistent with results from a cross-sectional study of Australian women and men [16] showing that active commuting was not associated with depression. In addition, a cross-sectional study of Estonian women also reported no association between active commuting (walking and cycling to work) and depressiveness [17]. The present findings together with previous studies [16, 17] suggest that commuting physical activity may not help in preventing depressive symptoms.

Several physiological mechanisms have been hypothesized for the inverse association of physical activity with risk of depression. Exercise may increase endorphin levels and the availability of neurotransmitters (e.g., dopamine, noradrenaline, and serotonin) [34]. A recent study of mice [35] showed that exercise-induced skeletal muscle peroxisome proliferator-activated receptor gamma coactivator 1 alpha modulated kynurenine metabolism, a key pathway of depression [36], and protected against stress-induced depression. Another recent study of mice [37] reported that exercise-induced adiponectin had neurogenic effects, resulting in reduced depression-like behaviors. In addition, exercise has been shown to induce hippocampal neurogenesis [38] and many trophic factors including brain-derived neurotrophic factor [39], effects that are possibly mediated by reduced inflammation [38]. Alternatively, psychological mechanisms including distraction from negative thoughts, enhancement self-esteem [34], experience of mastery, and social reinforcement [40] may explain the differential associations according to domains of physical activity observed in our study.

Major strengths in the present study include a prospective study design with a large-scale population size and annual assessment of depressive symptoms. However, the limitations of this study should be discussed. First, the questions for depressive symptoms used in the present study were not validated against clinical depression, although the scores of the depressive symptoms are highly correlated with those of SDS [28], and the questions are similar to those of existing depression scales such as the CES-D [29] and SDS [30]. Second, the physical activity questionnaire used in the present study was not validated, but it is similar to validated and reproducible questionnaires [41, 42]. Third, although we adjusted for an array of potential confounders, residual confounding and unmeasured factors including diet might have affected the results. Fourth, the present study used only baseline data to assess physical activity. Although study participants may have changed their habitual physical activity during the follow-up term, this type of misclassification would lead the results toward null findings. Fifth, participants who were excluded from the present analysis had different characteristics from those who remained in terms of sex, depressive symptoms, and marital status. We cannot deny the possibility of bias due to the selective inclusion. Finally, study participated were workers of a large company and their majority was male. Therefore, caution should be exercised in generalizing our finding to non-working population, workers from small- to middle-sized companies, and female. Nonetheless, in a nationally representative sample in Japan [43], approximately 30 % of adults engaged in moderate-intensity exercise and approximately 10 % of adults engaged in vigorous-intensity exercise in the 2006 Japanese national survey [43], whereas approximately 40 % of workers engaged in moderate- to vigorous-intensity exercise in the present study. In addition, age-specific prevalence of smoking in the national survey [43] was similar to those in the present study. Furthermore, the prevalence of obesity (BMI ≥ 25 kg/m^2^) in the present study was similar to those of the national survey [43]. Although direct comparison between the two surveys may not be feasible due to different measurement, the present working population appears to have characteristics similar to those of the general population of Japan in terms of health-related lifestyles.

## Conclusion

In the present study on a cohort of Japanese workers, we found a U-shaped association between leisure-time exercise and risk of depressive symptoms with the lowest risk being observed around 15 MET hours per week of leisure-time exercise. Therefore, for example, 300 min per week of moderate-intensity exercise, 150 min per week of vigorous-intensity exercise, or equivalent dose of combined intensities may be encouraged for workers to prevent depression. Given that sedentary work is associated with a higher risk of developing depressive symptoms, health-enhancing physical activity interventions may be needed for individuals who engage in sedentary work to help prevent mental illness. Further cohort studies should examine the relationship between clinical depression and various domains of physical activity.

## Abbreviations

BMI: Body mass index
CES-D: Center for Epidemiologic Studies Depression Scale
CI: Confidence interval
HR: Hazard ratio
J-ECOH: Japan Epidemiology Collaboration on Occupational Health
MET: Metabolic equivalent
SDS: Self-Rating Depression Scale
WHO: World Health Organization
